# Influence of feeding a grass hay diet during the early stage of the fattening period on growth performance, carcass characteristics, and meat production in Japanese Black steers

**DOI:** 10.1111/asj.13139

**Published:** 2018-12-18

**Authors:** Masahiro Shibata, Yasuko Hikino, Kazunori Matsumoto

**Affiliations:** ^1^ Faculty of Applied Life Science Nippon Veterinary and Life Science University Musashino, Tokyo Japan; ^2^ Livestock Production and Wildlife Management Research Division NARO Western Region Agricultural Research Center Oda Shimane Japan

**Keywords:** beef productivity, carcass and meat characteristics, gene expression, grass hay, water‐holding capacity

## Abstract

The present study investigated the influence of feeding a large amount of grass hay to steers from the early to middle fattening period on growth, carcass characteristics, and meat characteristics. Steers were randomly divided into grass hay‐fed (GHF,* n* = 6) and concentrate‐fed (CF,* n* = 6) groups. The dressed weight of the GHF steers was lower than that of the CF steers, but the final body weight was not significantly different between the groups. The GHF steers had decreased subcutaneous fat and rib thickness compared with the CF steers. Lipid content, monounsaturated fatty acids, and drip loss in the muscles were lower in the GHF steers than in the CF steers. Furthermore, n‐3 polyunsaturated fatty acids were higher in the GHF steers compared with the CF steers. The GHF steers had lower body weight during the middle fattening stage, which may have occurred as a result of muscle growth suppression caused by increased *Myostatin* expression; an increase in daily gain during the finishing period may have occurred as a result of muscle growth activation caused by decreased *Myostatin* expression. Feeding steers a grass hay‐based diet during the early fattening period possibly maintains the quantitative productivity of beef similarly to feeding a concentrate‐based diet.

## 1 INTRODUCTION

Most beef cattle in Japan are generally finished indoors on a concentrate‐based diet throughout the fattening period until slaughter. However, the feed self‐sufficiency rate for livestock production in Japan is low among advanced nations because most concentrate diets depend on import from foreign countries (MAFF 2008). Several studies on the diversity of beef cattle production have investigated the potentially effective use of roughage, i.e., whole crop silage (Shibata, Hikino, Imanari, Matsumoto, & Yamamoto, 2016), grass silage (French, O'Riordan, et al., 2000), and forage (Schroeder, Cramer, Bowling, & Cook, 1980), instead of concentrate diets.

Our previous study showed that feeding steers a large amount of a rice whole crop silage (rWCS)‐based diet from 16 to 28 months of age maintained equal quantitative productivity of beef relative to feeding a concentrate‐based diet, which led to a decrease in intramuscular fat (Shibata et al., 2016). In contrast, steers fed a large amount of grass hay from the middle stage of the fattening period had a lower slaughter body weight (BW) and decreased fat accumulation in muscle compared with steers fed a concentrate‐based diet because of lower total digestible nutrients (TDN) intake; this led to decreased muscular fat content, monounsaturated fatty acid (MUFA) concentration and n‐6/n‐3 fatty acid ratio in muscles (Shibata et al., 2012). The recommended value of the n‐6/n‐3 ratio, used as an index for maintaining human health, is less than four (Department of Health 1994). Several reports have revealed that the n‐6/n‐3 ratio is less than four in pasture‐fed steers (Descalzo et al., 2005; Muramoto, Higashiyama, & Kondo, 2005) and grass silage‐fed steers (Warren et al., 2008). Additionally, drip loss, which is used as an index of water‐holding capacity, is lower in beef from the grass hay‐fed steers (Shibata et al., 2012), grass‐fed steers (French, O'Riordan, et al., 2000), and rWCS‐fed steers (Shibata et al., 2016) than from concentrate‐fed steers.

Rumen and lower digestive tract weight increase in calves fed a large amount of roughage compared with those fed a large amount of concentrate (Harrison, Warner, Sander, & Looslt, 1960; Yamada, Aoki, & Nakanishi, 2000); generally, feeding adequate roughage is important because the digestive tract includes a rumen that is still developing during the early stage of fattening. Furthermore, it is important to expand the use of self‐sufficiency roughage sources in ruminant animals to reduce dependence on import concentrate diets. Based on those results, we hypothesized that the time and duration of feeding roughage may affect growth performance and carcass characteristics.

Gene expression monitoring can be used to deduce biological reactions in the living body during the fattening period, which may provide useful information for developing fattening systems. Furthermore, the roles and functions of many genes have been elucidated. Myostatin is a negative regulator of skeletal muscle growth, and its function is implicated in both hyperplasia and hypertrophy of skeletal muscle (McPherron, Lawler, & Lee, 1997). Our previous study revealed that *Myostatin*,* myosin heavy chain*, and *CCAAT/enhancer binding protein α* expression were associated with protein and fat accumulation in the muscles of grass hay‐fed steers (Shibata et al., 2011).

The present study investigated the influence of feeding steers a large amount of grass hay from the early to middle stage of the fattening period on growth performance, carcass characteristics and meat characteristics. Furthermore, we examined the relationship between growth performance and *Myostatin* gene expression.

## 2 MATERIALS AND METHODS

All experimental procedures and the management of steers in this study were performed in according with the Animal Experimental Guidelines of NARO Western Region Agricultural Research Center (NARO/WARC) established by the Animal Experimental Committee, NARO/WARC. The Animal Experimental Committee, NARO/WARC approved the study (certification number 08‐SOSHIRYO‐07).

### 2.1 Animal management and diets

Twelve Japanese Black steers, 10 months of age (10.1 ± 0.05), which had been bred in the NARO/WARC, were randomly selected and divided into two groups: a grass hay‐fed group (GHF) and a concentrate‐fed group (CF). All steers were housed individually in a tie‐stall barn. The GHF group (*n* = 6) was fed an experimental diet that consisted of Italian ryegrass hay [49% TDN and 5.3% crude protein (CP) on a dry matter basis] *ad libitum* and 2–3 kg/day concentrate (flaked corn, flaked barley, wheat bran and soybean meal; 82% TDN and 12% CP on a dry matter basis) from 10 to 22 months of age (21.8 ± 0.05). After this experimental period, the GHF group was fed 8–9 kg/day concentrate and grass hay *ad libitum* until 28 months of age (28.3 ± 0.22). During this experimental period, the reduction in CP caused by the decrease in the concentrate diet was supplemented with soybean meal (81% TDN and 50% CP on a dry matter basis) in the experimental diet. The CF group (*n* = 6) was fed 1.5 kg/day grass hay and concentrate *ad libitum* from 10 to 28 months of age. One roughage source was used in the present study to prevent influence of roughage differences on results in the present study. The present study was performed without restricted feeding vitamin A throughout the fattening period. The steers were slaughtered at 28 months of age, and skeletal muscle tissues were obtained for analysis of meat characteristics. The steers were weighed every week to record BW. The TDN intake was calculated from feed intake and estimated TDN of each diet.

### 2.2 Sample collection

Skeletal muscle tissues from the *M*.* longissimus lumborum* (LL) and *M. semitendinosus* (ST) of both groups were obtained by biopsy at 19, 22 and 28 months of age for gene expression analysis. The biopsy procedure was as follows: the animal was locally anesthetized by intramuscular injection of xylazine (Bayer, Tokyo, Japan) and subcutaneous injection of lidocaine (AstraZeneca, Osaka, Japan). An incision was subsequently made in the skin overlying the LL and ST muscles (Shibata et al., 2006). All biopsy samples were rapidly frozen in liquid nitrogen and stored at −80°C until RNA extraction.

### 2.3 Carcass evaluation and sample preparation

Carcasses were kept at 0°C for 24 hr and then evaluated by dressing percentage and measuring the beef marbling standard (BMS), beef fat color standard (BFS), beef color standard (BCS), rib eye area, rib thickness, and subcutaneous fat thickness of the section between the sixth and seventh ribs according to the Japanese New Beef Carcass Grading Standards (JMGA 1988). Skeletal muscle tissue samples from the LL and ST muscles were obtained from the carcasses to analyze meat characteristics such as drip loss, cooking loss, and Warner–Bratzler (WB) shear force. The muscles were processed into 2.5‐cm thick steaks, vacuum‐packed, stored at 2°C for 2 or 30 days after slaughter and frozen at −80°C until analysis.

### 2.4 Meat characteristics

Muscle tissues were minced to determine the nutrient contents. CP was calculated by quantitative analysis of nitrogen using the Kjeldahl method with copper sulfate and potassium sulfate as catalysts (AOAC, 1990). Lipids were extracted with diethyl ether for 16 hr using a Soxhlet extractor (AOAC, 1990). To analyze the fatty acid composition in the muscle tissue, the extracted lipids were converted to fatty acid methyl esters with boron trifluoride–methanol complex solution and analyzed by gas chromatography (AOCS, 2005).

Steaks were thawed for 24 hr at 4°C, and then carefully dried using paper tissue. Drip loss was calculated as the difference in weight before and after storage (Muramoto et al., 2005). Following drip loss measurement, the samples were broiled on electric grills to an internal temperature of 70°C, and then wrapped in plastic to prevent desiccation and stored at 4°C for approximately 12 hr (Montgomery et al., 2001; Realini et al., 2005). Cooking loss was calculated from the difference in weight before and after cooking (Montgomery et al., 2001; Muramoto et al., 2005). Six cores (1.3 cm in diameter) were removed from each steak parallel to the longitudinal orientation of the muscle fibers (Montgomery et al., 2001; Realini et al., 2005). All cores were sheared using a WB shear force machine, and the peak shear force was recorded. In the present study, only drip loss and cooking loss were used as indices of water‐holding capacity.

### 2.5 RNA isolation, cDNA synthesis, and gene expression measurement

Total RNA was extracted from muscle tissues using the TRIzol reagent (Invitrogen, Carlsbad, CA, USA) according to the manufacturer's protocol. First strand complementary DNA (cDNA) was synthesized from 3 μg of total RNA using SuperScript II RNase H^−^ (Invitrogen) reverse transcriptase with oligo(dT) primers (Invitrogen).

After reverse transcription, analysis of *Myostatin* gene expression was performed by real‐time PCR using an ABI 7500 detection system (Applied Biosystems, Foster City, CA, USA). The first strand cDNA was diluted with deionized water and amplified using the TaqMan gene expression master mix (Applied Biosystems) with a gene‐specific TaqMan probe and primers (Table 1). The real‐time PCR reaction was initially carried out for 2 min at 50°C, followed by 10 min at 95°C, and then 50 cycles of 15 s at 95°C and 1 min at 60°C. The housekeeping gene glyceraldehyde‐3‐phosphate dehydrogenase (*GAPDH*) was used as a normalization control. The TaqMan probe and primers were designed using Primer Express (Applied Biosystems).

**Table 1:** Sequences of real‐time PCR primers used in this study^a^

Gene^b^	GenBank accession No.	Forward primer (5′ to 3′)	Reverse primer (5′ to 3′)	Product size, bp
*Myostatin*	AB076403	GGCCATGATCTTGCTGTAACCT	GCATCGAGATTCTGTGGAGTG	144
*GAPDH* b	U85042	TGACCCCTTCATTGACCTTCA	ACCCCAGTGGACTCCACTACAT	201

a All sequence data are from the bovine sequence.

b *GAPDH*: glyceraldehyde‐3‐phosphate dehydrogenase.

### 2.6 Statistical analyses

All data are presented as mean ± *SEM*. The GHF and CF group data were analyzed using one‐way analysis of variance (ANOVA) and a *post hoc* Fisher's probability least significant difference (PLSD) test. Serial changes in gene expression data were analyzed using repeated measures ANOVA. If ANOVA revealed significant differences, then each dataset was assessed with a Fisher's PLSD *post hoc* test. *p *<* *0.05 was considered statistically significant.

## 3 RESULTS

### 3.1 Growth performance and carcass characteristics

Table 2 shows the effects of feeding a large amount of grass hay on the growth performance and feed intake of steers. The BW at 22 months of age, which was the end of the experimental diet feeding period, of the GHF group was significantly lower than that of the CF group, but the final BW was not significantly different between the two groups. The daily gain (DG) during the experimental period (10–22 months) was significantly lower in the GHF group compared with the CF group. In contrast, the DG during the finishing period (22–28 months) was significantly greater in the GHF group than in the CF group. The sum of the TDN intake of the GHF group during the experimental period was significantly lower than that of the CF group, but the TDN intake during the finishing period was significantly higher in the GHF group compared with the CF group. Although there was no change in the feed efficiency between the two groups during the experimental period, the feed efficiency during the finishing period was higher in the GHF group than in the CF group.

**Table 2:** Growth performance and feed intake of steers

	GHF	CF
Body weight (BW), kg		
Initial (10 months)	301 ± 5.9	300 ± 6.2
Middle (22 months)	488 ± 7.6*	612 ± 13.4
Final (28 months)	664 ± 9.5	703 ± 14.9
Daily gain (DG), kg/day		
Experimental period (10–22 months)	0.51 ± 0.03*	0.84 ± 0.03
Finishing period (22–28 months)	0.82 ± 0.03*	0.50 ± 0.05
TDN^a^ intake, kg/day		
Experimental period (10–22 months)		
Grass hay	2.33 ± 0.08*	0.56 ± 0.04
Concentrate	1.50 ± 0.02*	5.29 ± 0.08
Grass hay + concentrate	3.83 ± 0.08*	5.85 ± 0.09
Finishing period (22–28 months)		
Grass hay	1.84 ± 0.10*	0.55 ± 0.05
Concentrate	5.12 ± 0.09	4.92 ± 0.21
Grass hay + concentrate	6.96 ± 0.07*	5.46 ± 0.21
Feed efficiency, BW gain/TDN intake		
Experimental period (10–22 months)	0.133 ± 0.006	0.144 ± 0.006
Finishing period (22–28 months)	0.118 ± 0.004*	0.093 ± 0.010

^a^TDN: total digestible nutrients. Values are expressed as mean ± *SEM*. *p <* *0.05.

Table 3 shows the effects of feeding a large amount of grass hay on the carcass characteristics. Although there was no significant difference in the final BW between the groups, the dressed weight of the GHF group was significantly lower than that of the CF group. Compared with the CF group, the GHF group had significantly decreased rib thickness, which is a positive evaluation in the Japanese New Beef Carcass Grading Standards. In contrast, compared with the CF group, the GHF group had significantly decreased subcutaneous fat thickness, which is a negative evaluation in the Japanese New Beef Carcass Grading Standards. However, there were no significant differences in the dressing percentage, rib eye area, BMS, BCS, and BFS between the two groups, which indicate that there was no change in the beef quality.

**Table 3:** Carcass characteristics of steers

	GHF	CF
Dressed weight, kg	395 ± 4.5*	435 ± 8.9
Dressing percentage, %	72.2 ± 0.34	72.0 ± 0.29
Rib eye area, cm^2^	43.2 ± 2.06	48.4 ± 2.80
Rib thickness, cm	5.72 ± 0.23*	7.22 ± 0.15
Subcutaneous fat thickness, cm	1.90 ± 0.24*	3.44 ± 0.43
Beef marbling standard (BMS), No.	3.8 ± 0.58	4.0 ± 0.55
Beef color standard (BCS), No.	4.2 ± 0.49	4.0 ± 0.55
Beef fat color standard (BFS), No.	2.8 ± 0.20	2.8 ± 0.20

Values are expressed as mean ± *SEM*. *p <* *0.05.

### 3.2 Meat characteristics

Table 4 shows the effects of feeding a large amount of grass hay on nutrient content, water‐holding capacity, and WB shear force in steer skeletal muscles. Compared with the CF group, the ST muscle of the GHF group showed a significant decrease in extracted lipid content and a significant increase in moisture content. In terms of water‐holding capacity, the drip loss in the ST muscle was significantly lower in the GHF group than in the CF group. A similar trend was observed in the drip loss of the LL muscle, but there was no significant difference between the two groups (*p *=* *0.083). Furthermore, the cooking loss of the GHF group was lower than that of the CF group in the LL muscle, but there was no significant difference between the groups (*p *=* *0.093). The WB shear force was not different between the two groups.

**Table 4:** Nutrient contents, water‐holding capacity, and Warner–Bratzler shear force in the *M*. *longissimus lumborum* (LL) and *M*.* semitendinosus* (ST) muscles of steers

	LL		ST	
	GHF	CF	GHF	CF
Crude protein, %	18.2 ± 0.57	17.4 ± 0.65	20.9 ± 0.16	20.8 ± 0.27
Extract lipid, %	20.0 ± 2.36	25.7 ± 2.34	5.72 ± 0.79*	8.30 ± 0.69
Moisture, %	60.5 ± 1.79	55.8 ± 1.78	71.8 ± 0.70*	69.4 ± 0.59
Water‐holding capacity				
Drip loss, %	2.51 ± 0.18#	3.22 ± 0.31	4.86 ± 0.43*	6.56 ± 0.42
Cooking loss, %	34.0 ± 0.81#	36.5 ± 1.02	40.3 ± 0.68	39.4 ± 1.05
Shear force, kg				
Day 2	2.22 ± 0.07	2.09 ± 0.06	3.66 ± 0.09	3.80 ± 0.18
Day 30	1.42 ± 0.04	1.37 ± 0.08	2.71 ± 0.08	2.47 ± 0.13

Values are expressed as mean ± *SEM*. *p <* *0.05, ^#^ *p *<* *0.10.

Table 5 shows the effects of feeding a large amount of grass hay on fatty acid composition in steer skeletal muscles. The fatty acid component in both muscles showed a significantly higher proportion of C16:0 and saturated fatty acids (SFAs) in the GHF group than in the CF group. C17:1, C18:1, and MUFAs in both muscles were significantly lower in the GHF group compared with the CF group. C18:3n‐3 polyunsaturated fatty acid (PUFA) was detected in both muscles of the GHF group, but it was not detected in the both muscles from two samples of the CF group. The n‐6/n‐3 ratio in either muscle was significantly lower in the GHF group than in the CF group.

**Table 5:** Fatty acid composition (%) of total extractable lipids in the *M*. *longissimus lumborum* (LL) and *M*.* semitendinosus* (ST) muscles of steers

	LL		ST	
	GHF	CF	GHF	CF
C14:0	3.02 ± 0.26	2.94 ± 0.11	2.20 ± 0.13	2.62 ± 0.15
C14:1	1.00 ± 0.19	1.12 ± 0.24	0.76 ± 0.13	1.26 ± 0.28
C15:0	0.28 ± 0.02	0.24 ± 0.02	0.28 ± 0.02	0.26 ± 0.02
C16:0	29.2 ± 0.87*	25.2 ± 0.59	27.7 ± 0.65*	25.1 ± 0.38
C16:1	4.02 ± 0.24	4.50 ± 0.31	4.26 ± 0.45	5.60 ± 0.64
C17:0	0.72 ± 0.04	0.70 ± 0.08	0.70 ± 0.05	0.64 ± 0.09
C17:1	0.58 ± 0.04*	0.74 ± 0.05	0.62 ± 0.04*	0.82 ± 0.07
C18:0	11.4 ± 0.68	9.90 ± 0.82	9.90 ± 0.78	8.12 ± 1.00
C18:1	44.4 ± 1.19*	48.8 ± 0.64	45.3 ± 0.89*	48.2 ± 0.46
C18:2(n‐6)	1.82 ± 0.13	2.28 ± 0.17	2.88 ± 0.35	2.90 ± 0.27
C18:3(n‐3)^b^	0.18 ± 0.02	0.10 ± 0.00	0.20 ± 0.00	0.15 ± 0.05
C20:1	0.26 ± 0.02	0.34 ± 0.05	0.18 ± 0.02	0.26 ± 0.02
C20:3(n‐6)	ND^a^	ND	0.30 ± 0.08	0.15 ± 0.03
C20:4(n‐6)	0.20 ± 0.06	ND	0.70 ± 0.18	0.32 ± 0.04
Unidentified	2.94 ± 0.17	3.14 ± 0.43	3.80 ± 0.35	3.74 ± 0.33
n‐6/n‐3	11.5 ± 1.51*	26.0 ± 1.00	13.5 ± 1.45*	28.3 ± 6.75
SFA^c^	44.6 ± 1.18*	39.0 ± 1.27	40.8 ± 0.85*	36.7 ± 0.71
MUFA^d^	50.3 ± 1.20*	55.5 ± 0.86	51.1 ± 1.33*	56.1 ± 0.80
PUFA^e^	2.16 ± 0.23	2.34 ± 0.20	4.30 ± 0.70	3.40 ± 0.34

Values are expressed as mean ± *SEM*. *p <* *0.05.

^a^ND: not detected.

^b^not detected in two samples from both muscles of the CF group.

^c^SFA: saturated fatty acid (sum of C14:0, C15:0, C16:0, C17:0, and C18:0).

^d^MUFA: monounsaturated fatty acid (sum of C14:1, C16:1, C17:1, C18:1, and C20:1).

^e^PUFA: polyunsaturated fatty acid (sum of C18:2, C18:3, C20:3, and C20:4).

### 3.3 Gene expression in skeletal muscle

**Figure 1:**
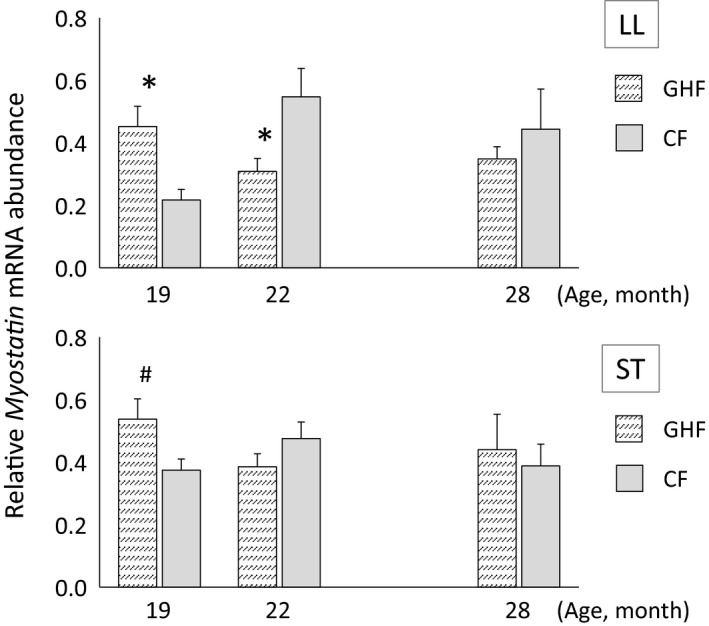
Developmental changes in *Myostatin* gene expression in the *longissimus lumborum* (LL) and *semitendinosus* (ST) muscles of Japanese Black steers assessed by real‐time PCR. The mRNA abundance of *Myostatin* was normalized to *GAPDH* mRNA. The data represent the mean ± *SEM* at each point. *p <* *0.05, #*p *<* *0.10 compared with the same age

The influence of feeding a large amount of grass hay on *Myostatin* gene expression in steer skeletal muscles is shown in Figure 1. *Myostatin* gene expression in the LL muscle at 19 months of age during the experimental period was significantly higher in the GHF group compared with the CF group. This gene's expression in the ST muscle of the GHF group was also greater than that in the ST muscle of the CF group at the same age, but there was no significant difference between the two groups (*p *=* *0.059). However, the *Myostatin* mRNA abundance in the LL muscle at 22 months of age was significantly lower in the GHF group compared with the CF group.

## 4 DISCUSSION

### 4.1 Growth performance and gene expression

The main aim of this study was to compare growth performance, carcass characteristics and meat characteristics between GHF and CF steers. Our previous study revealed that steers fed a large amount of rWCS‐based diet maintained equal quantitative productivity of beef relative to steers fed a concentrate‐based diet (Shibata et al., 2016). Steers fed grass silage *ad libitum* had a greater carcass weight than those fed a concentrate‐based diet (Warren et al., 2008). Those results showed that steer fattening was possible if mainly fed a roughage‐based diet. In contrast, steers fed grass hay *ad libitum* with restricted feeding of concentrate had less TDN intake, slaughter BW and dressed weight than those fed concentrate *ad libitum* (Muramoto, Aikawa, Shibata, & Nakanishi, 2002). Our previous study showed that slaughter BW, DG, and dressed weight decreased when steers were fed a large amount of grass hay compared with concentrate *ad libitum* after the middle stage of the fattening period, because there was decreased TDN intake (Shibata et al., 2012). In the present study, the middle BW was approximately 120 kg lighter in the GHF group than in the CF group. Although the final BW was also approximately 40 kg lighter in the GHF group than in the CF group, there was no difference between the groups. We suggest that these differences of BW gains during the two periods are due to increased TDN intake during the finishing period in the GHF group. Furthermore, in the present study, grass hay was fed from the early to middle stage of the fattening period (10–22 months of age). In contrast, in a previous study (Shibata et al., 2012), a large amount of grass hay was fed during the late stage of the fattening period (16–28 months of age). The results of both studies indicate that steers fed a large amount of grass hay may maintain a similar slaughter BW to those fed a concentrate‐based diet when they have adequate TDN intake and were fed the grass hay at the appropriate feeding time during the fattening period. For example, feeding grass hay during the early fattening stage can have a sufficient finishing period for BW gain.

Gene expression analysis can be used to predict information about the living body because the roles and functions of many genes have been elucidated. Natural mutations of the *Myostatin* gene are responsible for the double‐muscle phenotype of several steer breeds (Grobet et al., 1997; Kambadur, Sharma, Smith, & Bass, 1997; McPherron & Lee, 1997). Our previous studies showed that *Myostatin* gene expression increased when steers were fed a large amount of rWCS from the middle stage of the fattening period, which indicates that activation of skeletal muscle during the late fattening period of rWCS‐fed steers was due to decreased *Myostatin* gene expression (Shibata et al., 2016). In the present study, *Myostatin* gene expression in the LL and ST muscles at 19 months of age was higher in the GHF group compared with the CF group; this finding indicates that the skeletal muscle growth in the GHF group is suppressed around this time by *Myostatin*. In contrast, despite being measured before conversion from grass hay diet to concentrate diet, *Myostatin* expression in the LL muscle at 22 months of age was lower in the GHF group compared with the CF group; this finding suggests that skeletal muscle in the GHF group was activated by *Myostatin* at this time. Although the cause of *Myostatin* gene expression change is unknown despite continued feeding of the same diet, a similar expression pattern was observed in rWCS‐fed steers (Shibata et al., 2016). This change may be caused by diet acclimatization and/or the growth process of steers; however, this is speculation, and further study is necessary to clarify this phenomenon.

The present study suggests that the decreases in BW and DG during the middle fattening period in the GHF group may occur as a result of skeletal muscle growth suppression. In contrast, the gene expression patterns support the findings that the DG and feed efficiency during the finishing period were increased in the GHF group compared with the CF group. These findings suggest that compensatory growth may have started during the finishing period.

### 4.2 Carcass characteristics

A previous study reported that the DG and carcass weight of high‐roughage (alfalfa silage)‐fed steers were lower than those of high‐grain‐fed steers (Mandell, Buchanan‐Smith, & Campbell, 1998). Other studies have reported similar results (Muramoto et al., 2002; Shibata et al., 2012). The findings of those previous reports were consistent with our results of steers fed a large amount of roughage. However, there was no difference in final BW between the groups in the present study. A higher omasum weight has been observed in high‐grass hay‐fed steers compared with high‐concentrate‐fed steers (Muramoto et al., 2002). Several studies have reported that rumen and lower digestive tract weight increase in calves fed a large amount of roughage compared with those fed a large amount of concentrate (Harrison et al., 1960; Yamada et al., 2000). Mirzaei et al. (2015) suggested that physical stimulation is required for appropriate rumen development and growth performance of alfalfa hay‐fed dairy calves. Digestive tract weight generally increases in ruminant animals fed a large amount of roughage because this tract receives physical stimulation by roughage and the feed volume simply increases compared to feeding a concentrate‐based diet. Although the present study did not record the digestive tract weight of the steers, its weight may be different between the two groups. Our results indicate that the decrease in the dressed weight of the GHF group, despite the lack of change in the final BW between the two groups, may occur because of an increase in the digestive tract weight of the GHF group compared with the CF group. Further studies are needed to clarify the influence of rearing system on dressed weight.

The *longissimus* muscle area, marbling score and back fat decreased when steers were fed a high‐alfalfa silage diet compared with a high‐grain diet, but there was no change in the subcutaneous fat percentage (Mandell et al., 1998). The rib thickness and subcutaneous fat amount of the carcass body were lower in the grass hay‐fed steers than in the concentrate‐fed steers, whereas there was no difference in subcutaneous fat thickness (Muramoto et al., 2002). Generally, excessive subcutaneous fat is often considered inedible and discarded. Our previous studies reported lower rib thickness in grass‐fed (Shibata et al., 2012) and rWCS‐fed (Shibata et al., 2016) steers compared with that in concentrate‐fed steers. The decreases in subcutaneous fat thickness and rib thickness shown in the present study are consistent with the findings of previous reports in which steers were fed a large amount of roughage. Furthermore, reduction in subcutaneous fat thickness would reduce body fat accumulation and discarding rate, and lead to an increase in edible parts of a dressed carcass.

### 4.3 Meat characteristics

Our previous study showed that the CP content in the ST muscles of the grass hay‐fed steers was greater than that in the ST muscle of the concentrate‐fed steers (Shibata et al., 2012). In contrast, the CP content in the *semimembranosus* muscle of Angus steers was lower in grass‐fed steers than in concentrate‐fed steers (Srinivasan, Xiong, Blanchard, & Moody, 1998). Several previous reports have described no change in CP content in the *longissimus* muscle of forage‐fed Hereford steers (Schroeder et al., 1980), *longissimus dorsi* muscle of grass silage‐fed steers (French, O'Riordan, et al., 2000), and LL and ST muscles of rWCS‐fed Japanese Black steers (Shibata et al., 2016) compared with that in the muscles of concentrate‐fed steers. The present study showed no difference in the CP content in either muscle between the two groups. The analyzed muscles and breeds differed between this study and previous studies, but our results are consistent with those of many previous reports.

The lipid content in the *longissimus* muscle of grass silage‐fed Angus steers was greater than that of concentrate‐fed steers because silage‐fed steers had a larger amount of dry matter feed intake than concentrate‐fed steers (Warren et al., 2008). However, most previous reports showed decreased lipid content in the *longissimus* muscle of alfalfa silage‐fed Limousin steers (Mandell et al., 1998), LL, and ST muscles of grass hay‐fed Japanese Black steers (Shibata et al., 2012), *semimembranosus* muscle of grass‐fed Angus steers (Srinivasan et al., 1998) and ST muscle of rWCS‐fed Japanese Black steers (Shibata et al., 2016) compared with that in each of the muscles of concentrate‐fed steers. The present study showed decreased lipids in the ST muscle of the GHF group compared with that of the CF group, which is consistent with the findings of previous reports. These results indicate that steers fed a large amount of grass hay have decreased lipid deposition in their muscles compared with concentrate‐fed steers, but there was no influence on protein accumulation in the muscles.

In the present study, the increases in the fatty acid proportions of C16:0 and SFAs of the GHF group are consistent with the findings of previous reports in which steers were fed a grass silage diet (Warren et al., 2008) and a grass diet (Lehenska et al., 2008). Fatty acids in the LL and ST muscles exhibited lower proportions of C17:1, C18:1, and MUFAs in the GHF group than in the CF group, which corresponded to the findings of previous reports on barley silage‐fed steers (Nassu et al., 2011), grass hay‐fed steers (Shibata et al., 2012), grass‐fed steers (Lehenska et al., 2008; Srinivasan et al., 1998), and pasture‐fed steers (Muramoto et al., 2005). These results revealed that feeding steers a large amount of grass hay decreased the MUFA proportion and increased the SFA proportion. SFAs decreased as MUFAs increased in vitamin A‐nonsupplemented cattle, and the fatty acid desaturation index was lower in the vitamin A‐supplemented cattle than in the nonsupplemented cattle (Siebert et al., 2006). The present study did not assay for vitamin A and carotenoid concentration in the muscles, but they may have been obtained from grass hay and accumulated in the muscles.

The n‐3 PUFA reduces the risk of cardiovascular disease (Calder, 2004). The balance of n‐6/n‐3 ratio is an important determinant in decreasing the risk for coronary heart disease (Simopoulos, 2008). From 8 to 14 months and 19 and 24 months, decreased n‐6/n‐3 ratios and an increased total amount of n‐3 PUFAs were shown in Angus–Holstein crossbred steers that were fed a large amount of grass silage compared with steers that were fed concentrate (Warren et al., 2008). The n‐6/n‐3 ratios in continental crossbred steers fed concentrate, grass silage, and grass for 85 days were 4.15, 3.61, and 2.33, respectively; the lowest values were observed in the grass‐fed steers (French, Stanton, et al., 2000). The n‐6/n‐3 ratios of nonpregnant beef cows fed a large amount of grass hay and barley silage for 20 weeks were 3.32 and 4.39, respectively (Nassu et al., 2011). Several reports showed that the n‐6/n‐3 ratio in the skeletal muscle decreased in pasture‐fed steers compared with concentrate‐fed steers (Descalzo et al., 2005; Muramoto et al., 2005). Those previous reports revealed that the n‐6/n‐3 ratio decreases in grass hay‐, grass silage‐, barley silage‐, and pasture‐fed steers were due to increases in n‐3 PUFAs. Although the present study showed that the n‐6/n‐3 ratio was more than four in the GHF group, it was lower in the GHF group than in the CF group. These results suggest that the n‐6/n‐3 ratio improved in steers fed a large amount of grass hay compared with the concentrate‐fed steers, and that this ratio was slightly closer to the recommended value.

The drip loss and cooking loss of meat have an important influence on the water‐holding capacity of meat. Previous reports demonstrated that there was no difference in drip loss between concentrate‐fed steers and either alfalfa silage‐fed steers (Mandell et al., 1998) or grazing steers (Dufrasne, Gielen, Limbourg, Eenaeme, & Istasse, 1995). In contrast, drip loss was lower in steers fed a large amount of grass hay during the late fattening stage compared with concentrate‐fed steers (Shibata et al., 2012). Drip losses in continental crossbred steers fed concentrate, grass silage and grass for 85 days were 38.1, 29.5, and 26.4, respectively; the lowest values were obtained from the grass‐fed steers (French, O'Riordan, et al., 2000); those results are consistent with our results. Furthermore, our previous study revealed decreased drip loss in the muscle of steers fed a large amount of rWCS, which was due to stabilization of cell membranes by high amounts of the antioxidant vitamins α‐tocopherol, retinol, and β‐carotene, which are present in rWCS (Shibata et al., 2016). The α‐tocopherol and β‐carotene contents in the muscle of pasture‐fed steers were higher than those of concentrate‐fed steers, and pasture finishing decreased drip loss of the beef (Muramoto et al., 2005). High α‐tocopherol in meat effectively reduces drip loss in Holstein steers (Mitsumoto, Arnold, Schaefer, & Cassens, 1995). Although we did not analyze the antioxidant vitamin concentrations in the muscles, we suspect that these concentrations are increased in the muscles of steers fed a large amount of grass hay because they intake grass hay‐derived vitamins. Several reports have shown no change in cooking loss between concentrate‐fed steers and alfalfa silage‐fed steers (Mandell et al., 1998) or grass‐fed steers (French, O'Riordan, et al., 2000). However, higher cooking loss has been reported in beef from grazing steers compared with concentrate‐fed steers (Dufrasne et al., 1995). In contrast, our previous report demonstrated that cooking loss tended to be lower in the grass hay‐fed steers than in the concentrate‐fed steers (Shibata et al., 2012). Although the cooking loss results slightly varied because of differences in the rearing system used in previous reports, the results of the present study are consistent with the findings of our previous report in which the steers were reared in a similar environment (Shibata et al., 2012). The drip loss and cooking loss results suggest that the water‐holding capacity of beef improved in steers that were kept indoors fed a large amount of grass hay.

## 5 CONCLUSION

The present study revealed that feeding a largely grass hay‐based diet to steers until the middle stage of the fattening period potentially produces a similar quantitative productivity of beef to feeding a concentrate‐based diet. Although middle BW was lower in the GHF group than in the CF group, there was no difference in the final BW between the groups, which suggests that compensatory growth may have started during the late fattening period in the GHF group. In terms of meat quality, the present study suggested that water‐holding capacity improved in steers fed a large amount of grass hay because there was decreased drip loss and cooking loss. Furthermore, improvement in the n‐6/n‐3 ratio due to an increase in n‐3 fatty acids was observed in grass hay‐fed steers, which indicates that this ratio was closer to the recommended value.
